# Cardiac Hydatid Cyst: A Diagnostic and Therapeutic Challenge Requiring a Multidisciplinary Approach

**DOI:** 10.1016/j.jaccas.2026.106857

**Published:** 2026-02-04

**Authors:** Marta Alcalá Ramírez del Puerto, Pedro Fernández Martín, Germán Merchán Ortega, Joaquín Alberto Cano Nieto, Antonio Plata Ciezar, Esteban Sarria Garcia, Cristobal Urbano Carrillo

**Affiliations:** a Department of Cardiology, Hospital Regional Universitario de Málaga, Málaga, Spain; b Department of Infectious Diseases, Hospital Regional Universitario de Málaga, Málaga, Spain; c Department of Cardiovascular Surgery, Hospital Regional Universitario de Málaga, Málaga, Spain

**Keywords:** cardiac magnetic resonance, echocardiography, imaging, left ventricle, thoracic, treatment

## Abstract

**Background:**

Cardiac hydatid cysts are a rare manifestation of echinococcosis that can impair ventricular function and pose significant diagnostic and therapeutic challenges. Early recognition and multidisciplinary management are crucial to avoid life-threatening complications.

**Case Summary:**

A 33-year-old man from Morocco presented with progressive dyspnea, atypical chest pain, and constitutional symptoms. Echocardiography and cardiac magnetic resonance imaging revealed a multiloculated cystic lesion with daughter vesicles in the left ventricle, consistent with a hydatid cyst. Surgical excision via left ventriculotomy and postoperative albendazole therapy led to full clinical recovery.

**Discussion:**

Isolated cardiac hydatidosis without extracardiac involvement is exceedingly rare and often presents with nonspecific symptoms. Multimodal imaging was key for diagnosis and surgical planning. Combined medical and surgical treatment with perioperative precautions ensured an excellent outcome.

**Take-Home Messages:**

Cardiac hydatid cysts, although exceptional, should be suspected in patients from endemic regions presenting with intracardiac masses. Negative serologic results do not exclude the diagnosis. Echocardiography and cardiac magnetic resonance imaging are key for diagnosis and surgical planning. Long-term follow-up is mandatory because of the risk of recurrence, even after complete resection.

## Visual Summary

**Figure:**
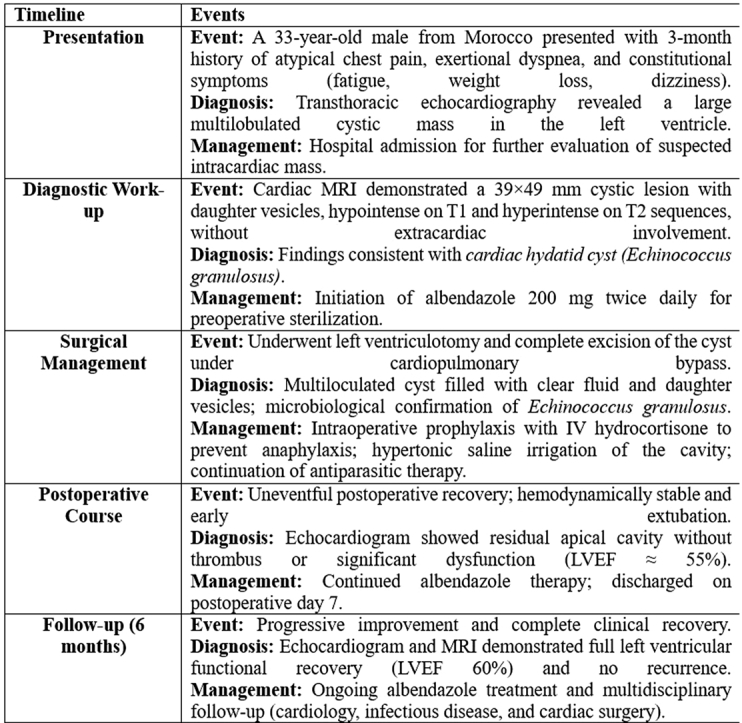

## Presentation History

We report the case of a 33-year-old man, originally from Morocco and residing in Spain for the past 15 months, who presented with a 3-month history of chest pain radiating to the throat, not clearly related to exertion. The pain was associated with progressive dyspnea on minimal exertion, intermittent sensations of thermal dysregulation without documented fever, and episodes of dizziness with vertigo. He also reported unquantified weight loss and close contact with animals, particularly dogs, during childhood. Transthoracic echocardiography performed in an outpatient setting revealed an intracardiac multiloculated mass in the left ventricle, prompting hospital admission.

**Visual Summary:**
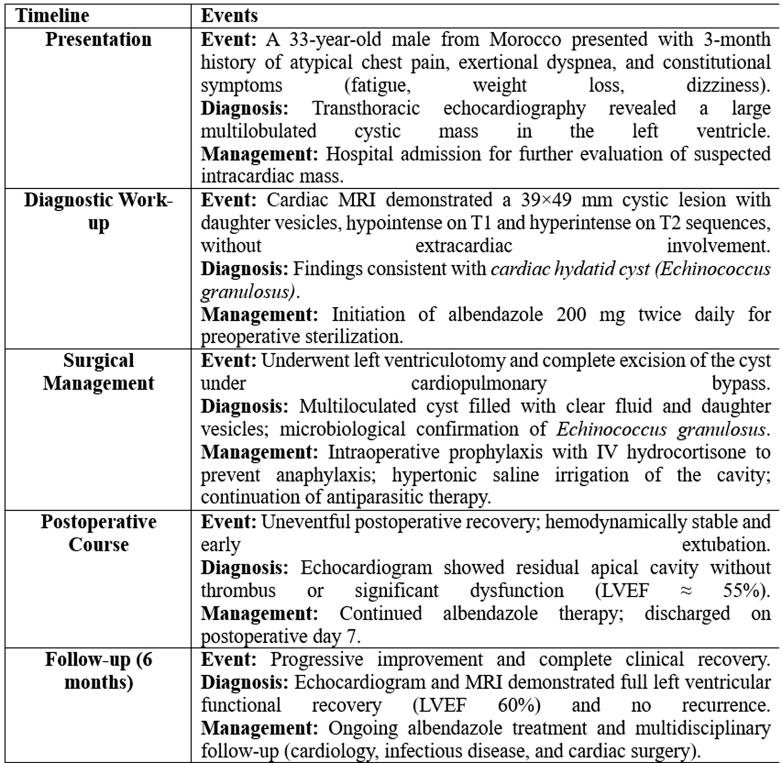
Clinical Timeline of a Left Ventricular Cardiac Hydatid Cyst Summary of the presentation, diagnostic evaluation, surgical treatment, postoperative course, and 6-month follow-up. IV = intravenous; LVEF = left ventricular ejection fraction; MRI = magnetic resonance imaging.

## Past Medical History

The patient had no relevant past medical history. He had no known drug allergies, no toxic habits, was not on any medication, and had no family history of cardiac disease.

## Differential Diagnosis

In a young patient presenting with chest pain, dyspnea, and an intraventricular mass, differential diagnosis should include cardiac tumors (benign or malignant), intraventricular thrombi, infective endocarditis with vegetations or abscesses, and cystic lesions such as a hydatid cyst. Thrombus formation is usually associated with ventricular dysfunction or a prothrombotic state, neither of which were present in this case. Cardiac tumors, although possible, often exhibit different echocardiographic characteristics and clinical behavior. The combination of clinical history, imaging findings, and epidemiological background guided the diagnostic reasoning toward a parasitic etiology.

## Diagnostic Work-Up

**Figure 1:**
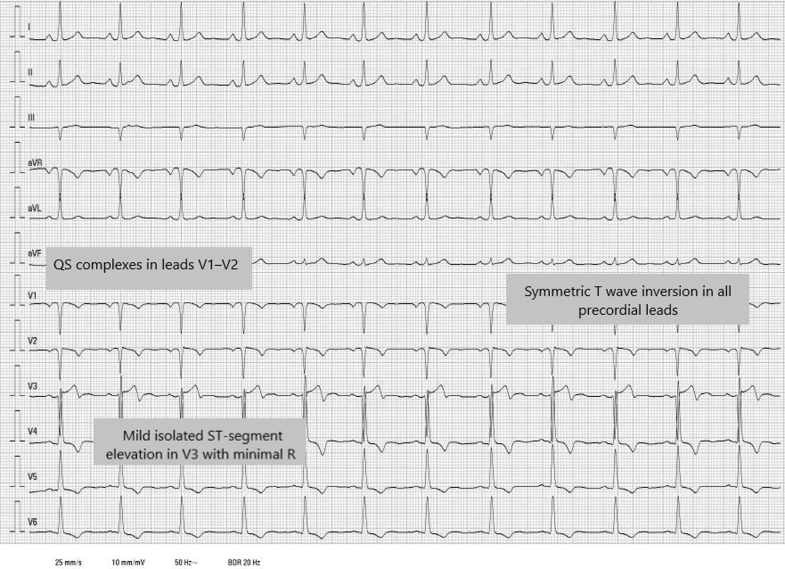
12-Lead Electrocardiogram on Admission Showing Sinus Rhythm at 75 beats/min, a Normal PR Interval, and Narrow QRS Complexes QS complexes are present in leads V_1_ and V_2_, with a minimal R-wave in lead V_3_. A symmetric T-wave inversion is seen in all precordial leads, along with mild, isolated ST-segment elevation in lead V_3_.

A 12-lead electrocardiogram (Figure 1) showed sinus rhythm at 75 beats/min with a normal PR interval and narrow QRS complexes. QS complexes were present in leads V_1_ and V_2_, with a minimal R wave in lead V_3_, and symmetrical T-wave inversion in all precordial leads, along with mild, isolated ST-segment elevation in lead V_3_.

**Figure 2:**
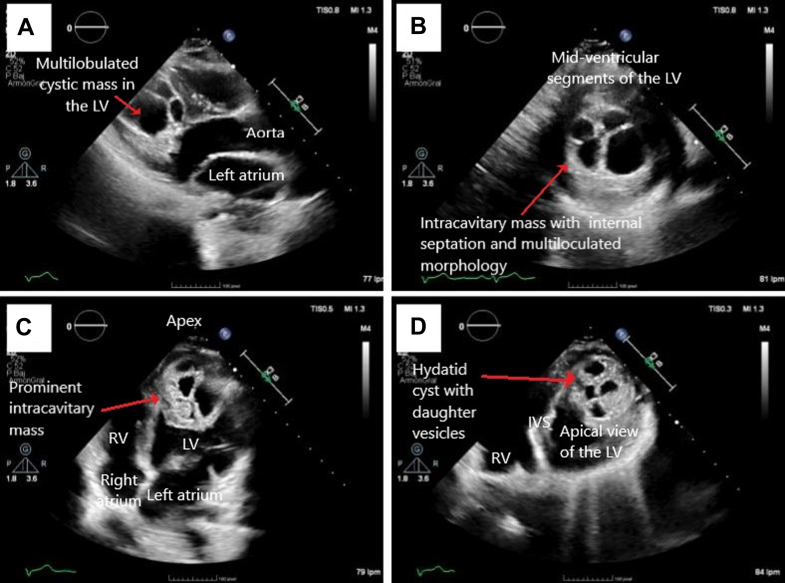
Transthoracic Echocardiographic Images Showing a Large Multiloculated Cystic Intracavitary Mass in the LV, Suggestive of a Hydatid Cyst With Daughter Vesicles (A) Parasternal long-axis view showing a heterogeneous cystic lesion confined to the apical and mid-level regions of the LV. (B) Parasternal short-axis view at the mid-ventricular level, showing internal septation and multiloculated morphology. (C) Apical 4-chamber view with a prominent intracavitary mass extending toward the apex. (D) Modified apical 4-chamber view centered on the apex, clearly delineating the extent of the cystic lesion. IVS = interventricular septum; LV = left ventricle; RV = right ventricle.

Transthoracic echocardiography (Figure 2, Video 1) revealed a large multiloculated cystic mass measuring 40 × 43 mm occupying the apical and mid-left ventricular cavity. The mass contained hyperechogenic internal components compatible with daughter vesicles, suggesting a hydatid cyst. Left ventricular ejection fraction (LVEF) was moderately reduced, whereas the right heart chambers and valves were structurally and functionally normal. No hepatic cysts were seen during the abdominal sweep.

**Figure 3:**
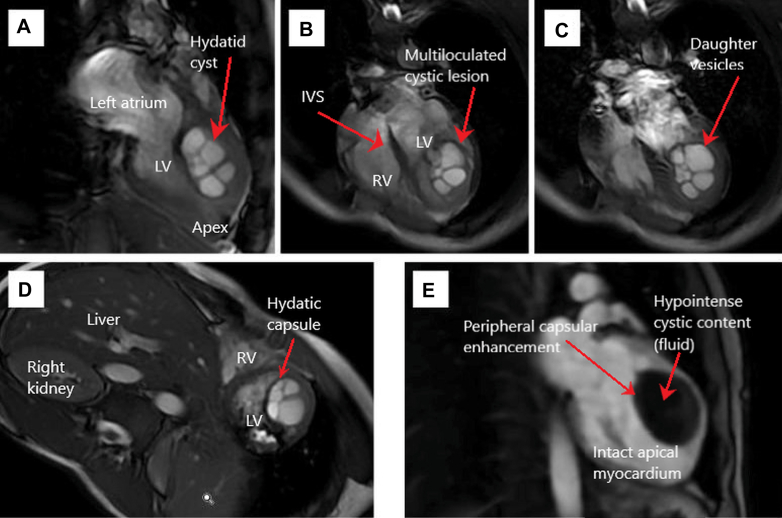
Cardiac Magnetic Resonance Imaging Cine-SSFP sequences in (A) sagittal and (B to D) axial planes show a multiloculated cystic lesion in the apex of the LV, containing multiple daughter vesicles—an imaging feature highly suggestive of an active hydatid cyst. (E) T1-weighted sequence with late gadolinium enhancement in the sagittal view demonstrates a unilocular cystic lesion with homogeneously hypointense content (consistent with fluid) and peripheral capsular enhancement, located in the apical region of the LV. This sequence allows assessment of capsular integrity and helps rule out myocardial infiltration or rupture. SSFP = steady-state free precession; other abbreviations as in Figure 2.

Cardiac magnetic resonance imaging (MRI) (Figure 3) confirmed an intramyocardial and intracavitary mass in the anteromedial and anteroapical segments of the left ventricle, measuring approximately 39 × 49 mm. The lesion appeared hypointense on T1-weighted and hyperintense on T2-weighted sequences, with internal daughter cysts. There was no early contrast uptake, but late enhancement of the capsule was observed. LVEF was moderately decreased (36%), without evidence of myocardial edema or fibrosis.

**Figure 4:**
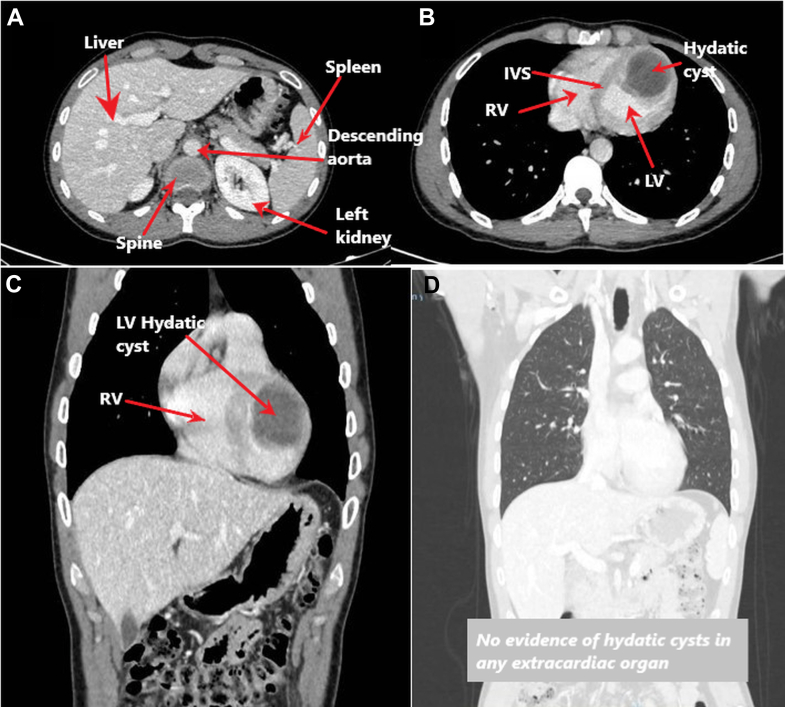
Preoperative Contrast-Enhanced CT Demonstrating Isolated Cardiac Hydatid Disease (A) No mediastinal lymphadenopathy, pulmonary nodules, or parenchymal abnormalities are identified. (B-C) Preoperative contrast-enhanced CT of the chest and abdomen showing a 40 x 36 mm multiloculated cystic mass located in the LV wall, radiologically consistent with a cardiac hydatid cyst. (D) Abdominal imaging reveals normal liver, gallbladder, biliary tree, pancreas, spleen, kidneys, adrenal glands, and mesenteric structures, with no evidence of hydatid cysts in any extracardiac organ. These findings confirm isolated cardiac hydatidosis with no other systemic involvement. CT = computed tomography; other abbreviations as in Figure 2.

A thoracoabdominal computed tomography (CT) scan corroborated the diagnosis, confirming a left ventricular hydatid cyst without extracardiac involvement (Figure 4). Laboratory studies showed normal blood count and biochemistry, without eosinophilia. Inflammatory markers were negative, as were serologies for *Echinococcus granulosus* and *Taenia solium*. QuantiFERON-TB (Qiagen) test and blood and stool cultures were negative, and the coagulation profile was normal.

The absence of fever, systemic infection, or prior cardiac disease made endocarditis unlikely. The multiloculated cystic nature of the mass, along with the epidemiological history of dog exposure in an endemic region, strongly supported the diagnosis of a cardiac hydatid cyst.

## Management

Upon suspicion of a cardiac hydatid cyst, antiparasitic therapy with albendazole 200 mg twice daily was initiated immediately and continued for 7 days preoperatively to reduce the risk of intraoperative parasitic dissemination and to sterilize the lesion. After completing the extension study (thoracoabdominal CT and serologies), and in the absence of extracardiac involvement, scheduled urgent surgery was performed because of the high risk of mechanical or embolic complications.

**Figure 5:**
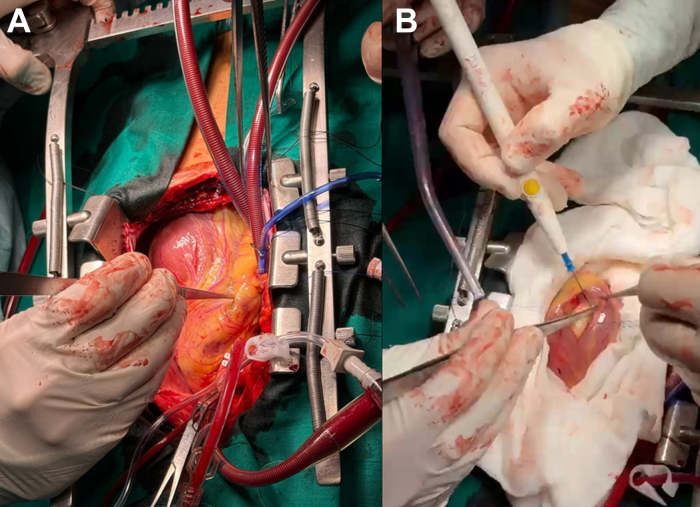
Intraoperative Views During Surgical Treatment of Cardiac Hydatidosis (A) Epicardial exposure showing the apical imprint of the hydatid cyst on the left ventricular apex, located lateral to the left anterior descending coronary artery system. (B) Cyst delimitation and controlled opening, with gauze pads soaked in hypertonic saline placed circumferentially around the operative field to inactivate any potentially escaped daughter vesicles and prevent dissemination.

Complex cardiac surgery was performed via left ventriculotomy for complete excision of the cyst (Figure 5, Video 2). A perioperative safety protocol for hydatid disease was activated, including preinduction intravenous hydrocortisone to prevent anaphylactic reactions in case of cyst rupture and antigen release. The cyst was multiloculated, filled with clear fluid and daughter vesicles consistent with *E granulosus*, later confirmed by microbiological analysis. The cavity was drained, the daughter cysts removed, and the area irrigated with hypertonic saline for scolicidal sterilization (Video 3). The procedure was uneventful, and the patient was transferred to the intensive care unit postoperatively. Antibiotic prophylaxis and albendazole therapy were continued.

The postoperative intensive care unit course was favorable. The patient remained hemodynamically stable, without arrhythmias or infectious complications, and was extubated early. He was transferred to the general ward on postoperative day 4. Clinical evolution was stable, without signs of heart failure. Echocardiographic follow-up revealed a residual apical cavity with possible intracavitary flow, pending further evaluation. Albendazole therapy was continued on an outpatient basis, with the aim of completing at least 6 months of antiparasitic treatment. Serologies remained negative, likely because of isolated intracardiac involvement with limited systemic sensitization.

## Evolution and Follow-Up

The patient showed progressive clinical improvement with no hemodynamic or infectious complications. Echocardiographic evaluations of control patient demonstrated full recovery of ventricular function (LVEF 60%) and anatomical stability. Residual apical dyskinesia was observed, consistent with postsurgical changes (Video 4). Albendazole treatment was maintained, and multidisciplinary follow-up is ongoing.

## Discussion

Hydatidosis (cystic echinococcosis) is a zoonotic infection caused by the larval stage of *E granulosus*. It is endemic in temperate regions such as Eurasia, Australia, South America, parts of Africa, and the Mediterranean basin.^1^ In Spain, active foci persist in rural regions such as Navarra, La Rioja, and Castilla y León, associated with extensive livestock farming, close contact with dogs, and poor waste management. Our patient had an epidemiological profile compatible with this exposure risk.

The parasite's life cycle involves dogs and other canids as definitive hosts and herbivores—and occasionally humans—as intermediate hosts. Ingested eggs release embryos that penetrate the intestinal mucosa and spread hematogenously, primarily to the liver and lungs.^2^ In rare instances, embryos reach the heart via the coronary arteries. Cardiac involvement is extremely rare, accounting for 0.02% to 2% of all hydatid disease cases, and typically presents as a solitary intramyocardial cyst, most commonly in the left ventricle.^3^ Our case, involving a large apical cyst with no extracardiac involvement, is highly unusual.

Cardiac hydatid cysts may remain asymptomatic for years, growing at an estimated rate of 1 cm/y. They eventually produce symptoms depending on size and location, including chest pain, dyspnea, palpitations, or syncope. In our case, the presentation included nonspecific symptoms such as fatigue, chest pain, and exertional dyspnea over several weeks. Complications include cyst rupture with anaphylaxis, cardiac tamponade, systemic embolism, myocardial ischemia from coronary compression, and conduction abnormalities.^4^ Despite the absence of complications at diagnosis, the risk of fatal progression justified early surgical intervention.

Diagnosis requires a high index of suspicion, especially in isolated cardiac forms with negative serology, as in this case. Cardiac MRI is the gold standard for lesion characterization, revealing the multivesicular appearance with daughter cysts. Echocardiography and CT provide complementary anatomical information. Electrocardiographic findings are often nonspecific, including T-wave inversions or bundle branch blocks, as seen in our patient. Although serological testing has high specificity, results may be negative in localized or encapsulated disease.^3^

The treatment of choice is surgical resection combined with antiparasitic therapy. Unlike hepatic or pulmonary hydatidosis, which may allow conservative management, cardiac involvement requires surgical excision even in inactive stages because of the high risk of complications.^5^ In this case, partial cystopericystectomy with hypertonic saline irrigation was performed under cardiopulmonary bypass. Albendazole therapy was initiated at diagnosis and continued postoperatively. Albendazole is the antiparasitic agent of choice, reducing cyst viability and recurrence risk. Given the potential for intraoperative anaphylaxis, preoperative hydrocortisone prophylaxis was administered—as is standard in high-risk hydatid operations.

Prognosis depends on early diagnosis, cyst location, and surgical complexity. Reported mortality is approximately 11.1%.^6^ Local recurrence can occur even 9 years posttreatment, warranting clinical, serological, and imaging surveillance for at least a decade.^6^ This case underscores the need to consider hydatidosis in the differential diagnosis of intracardiac masses, even without eosinophilia or positive serology, particularly in patients from endemic areas.

## Conclusions

This case illustrates a rare isolated presentation of cardiac hydatidosis affecting only the left ventricle, without hepatic or pulmonary involvement, and with a nonspecific clinical profile that initially hindered diagnosis. Transthoracic echocardiography provided the first clue, whereas cardiac MRI was essential in identifying the mass as a nonenhancing, multiloculated cystic lesion highly suggestive of a hydatid cyst. Thoracoabdominal CT ruled out extracardiac disease, and the diagnosis was confirmed intraoperatively and by microbiology.

Management of this uncommon condition requires early co-ordinated multidisciplinary intervention. Delayed elective surgery after albendazole therapy, along with specific anesthetic precautions (steroid prophylaxis to prevent anaphylaxis), allowed complete resection without major complications.

This case highlights the importance of including hydatid disease in the differential diagnosis of intracardiac masses in patients from endemic regions, even in the absence of eosinophilia or positive serology, and underscores the value of multimodal imaging in diagnosis and treatment planning.

### Patient consent

The authors confirm that written informed consent was obtained from the patient for publication of this case report and accompanying images. All identifying information has been removed to ensure anonymity.

### Data Availability

All data relevant to the case are included in the article and its figure legends. Additional details are available from the corresponding author upon reasonable request.

**Take-Home Messages**

• Cardiac hydatid cysts, although exceptional, should be suspected in patients from endemic regions presenting with intracardiac masses.
• Negative serology does not exclude the diagnosis.
• Echocardiography and cardiac magnetic resonance imaging are key for diagnosis and surgical planning.
• Long-term follow-up is mandatory because of the risk of recurrence, even after complete resection.

## Funding Support and Author Disclosures

The authors have reported that they have no relationships relevant to the contents of this paper to disclose.

## Appendix

Video 1 Initial Transthoracic Echocardiogram (Apical 4-Chamber View, Cine Loop) Showing a Large Multiloculated Cystic Mass Occupying the Apical and Mid Segments of the Left Ventricular Cavity The lesion exhibits heterogeneous echogenic content with visible internal septations, consistent with daughter vesicles of a hydatid cyst. Left ventricular systolic function appears moderately reduced.

Video 2 Intraoperative Footage of Cardiac Surgery Showing Complete Excision of a Multiloculated Hydatid Cyst From the Left Ventricle Via Left Ventriculotomy The cystic cavity is opened, revealing clear fluid and multiple daughter vesicles. Surgical debridement and irrigation with hypertonic saline are performed to ensure sterilization. No intraoperative complications occurred.

Video 3 Final Irrigation of the Residual Cystic Cavity With Hypertonic Saline After Evacuation of Daughter Vesicles Hypertonic saline is instilled directly into the opened cavity to achieve chemical sterilization, inactivating any remaining scolices or microscopic parasitic debris and reducing the risk of intraoperative dissemination or recurrence.

Video 4 Follow-Up Transthoracic Echocardiography From the Patient's Most Recent Evaluation Showing Preserved Biventricular Systolic Function and Postsurgical Apical Changes, With a Retracted Residual Cyst Capsule and Localized Apical Dyskinesia The transient LV systolic dysfunction present before and shortly after surgery had recovered at this evaluation.
